# Supplementary figures and images for: FAK loss reduces BRAFV600E-induced ERK phosphorylation to promote intestinal stemness and cecal tumor formation (part 1 of 2)
Source: eLife. 2024 Jun 26;13:RP94605. doi: 10.7554/eLife.94605 (PMC11208045; doi:10.7554/eLife.94605)

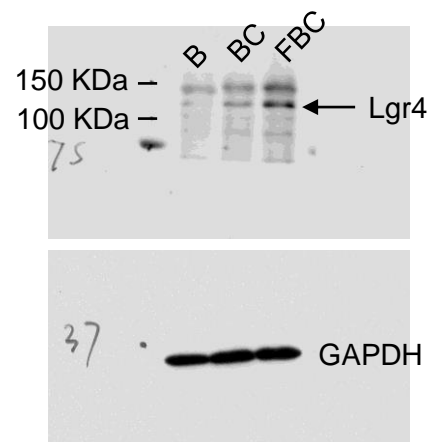

Supplement: Figure 4—source data 1. [file elife-94605-fig4-data1.pdf]

B BC ABC

CLIP 1211

(For Lgr4)

37

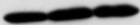

from same

member

Supplement: Figure 4—source data 2. [file elife-94605-fig4-data2.zip › Figure 4-source data 1 Raw unedited gels for figure 4.pdf]

B Bc F13C

75

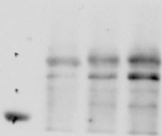

Supplement: Figure 4—source data 2. [file elife-94605-fig4-data2.zip › Figure 4-source data 2 Raw unedited gels for Figure 4.pdf]

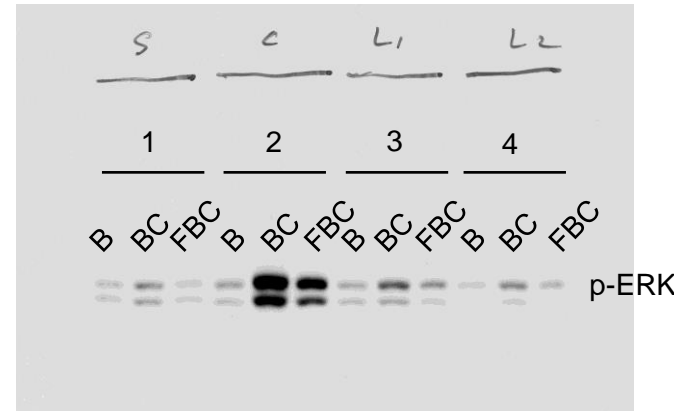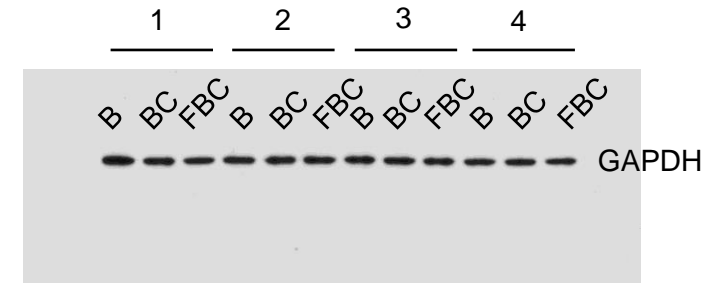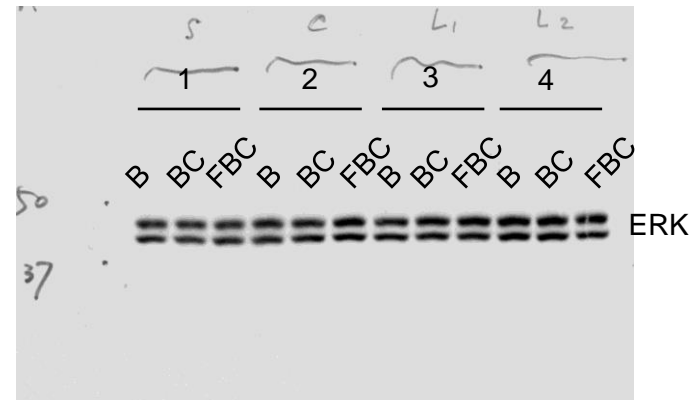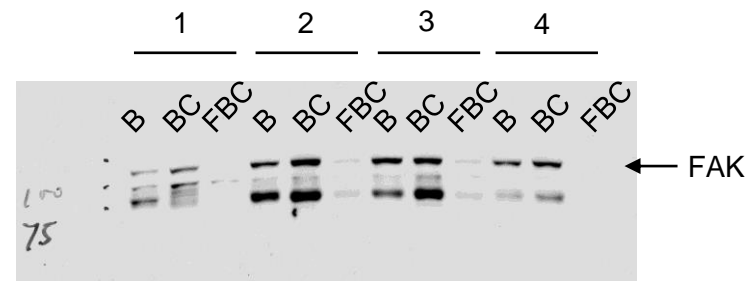

Supplement: Figure 5—source data 1. [file elife-94605-fig5-data1.zip › Figure 5-Source data 1 Uncropped and labeled gels for Figure 5.pdf]

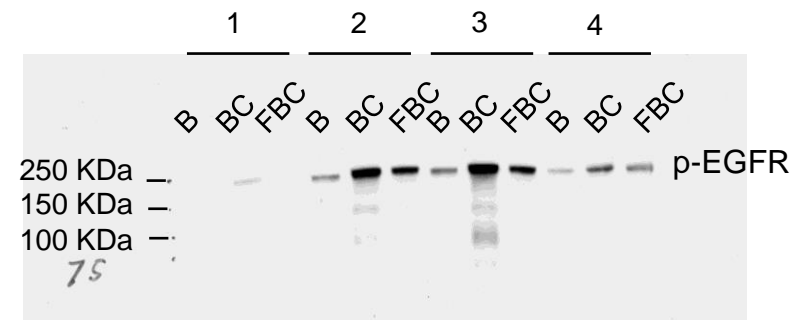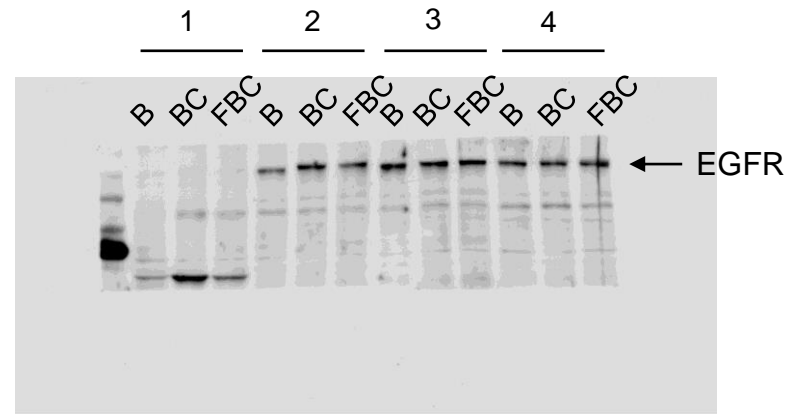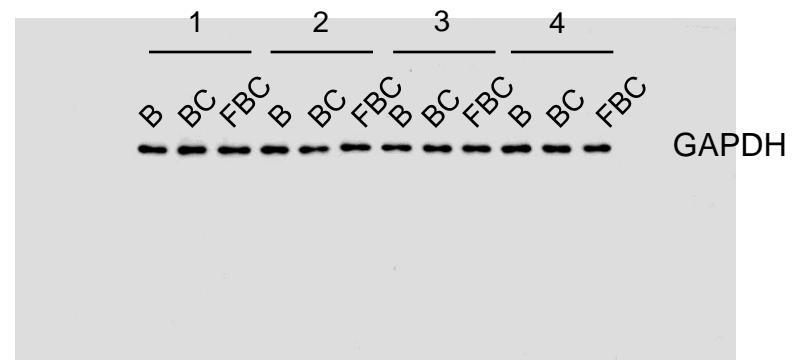

Supplement: Figure 5—source data 1. [file elife-94605-fig5-data1.zip › Figure 5-Source data 2 Uncropped and labeled gels for Figure 5.pdf]

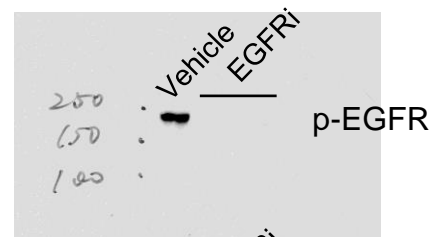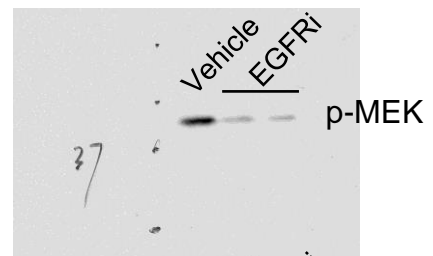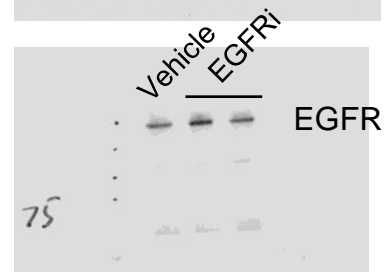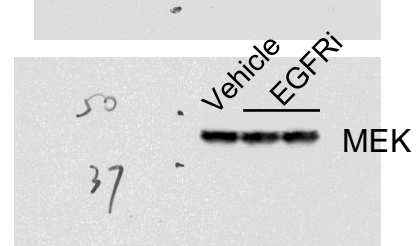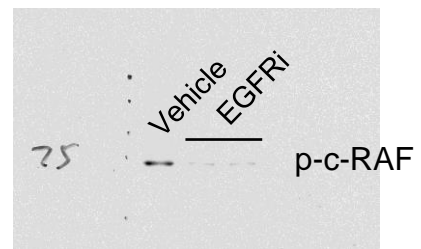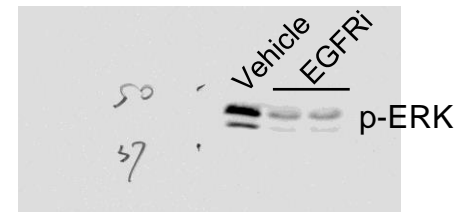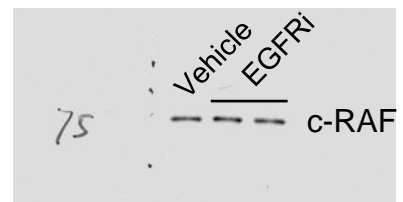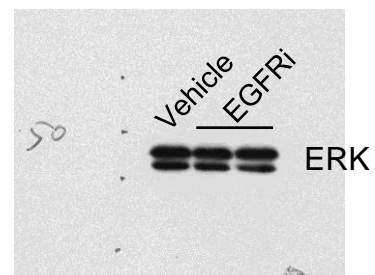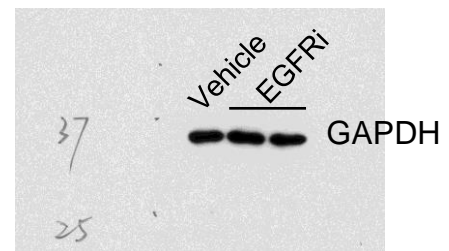

Supplement: Figure 5—source data 1. [file elife-94605-fig5-data1.zip › Figure 5-Source data 3 Uncropped and labeled gels for Figure 5.pdf]

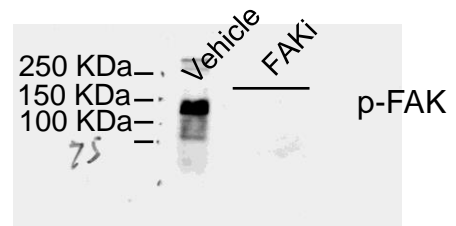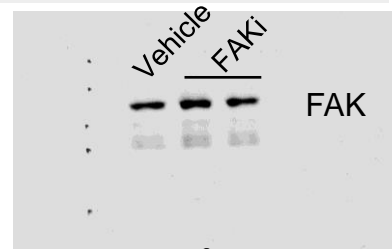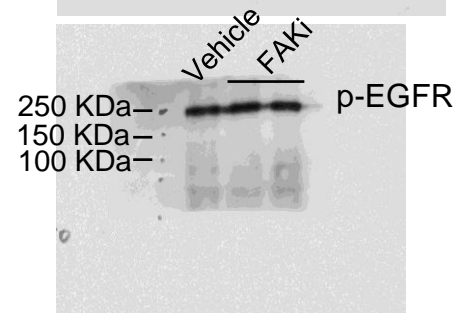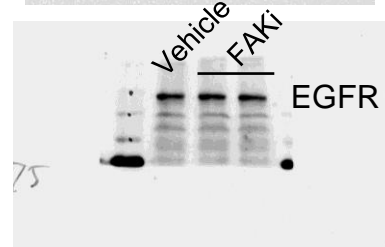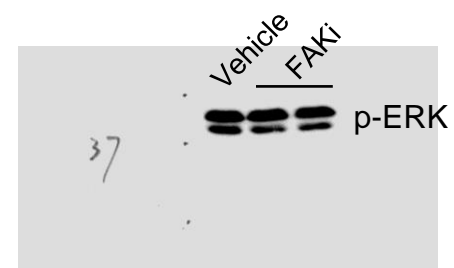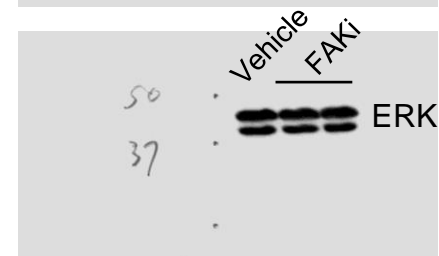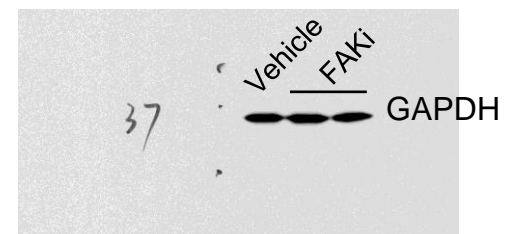

Supplement: Figure 5—source data 1. [file elife-94605-fig5-data1.zip › Figure 5-Source data 4 Uncropped and labeled gels for Figure 5.pdf]

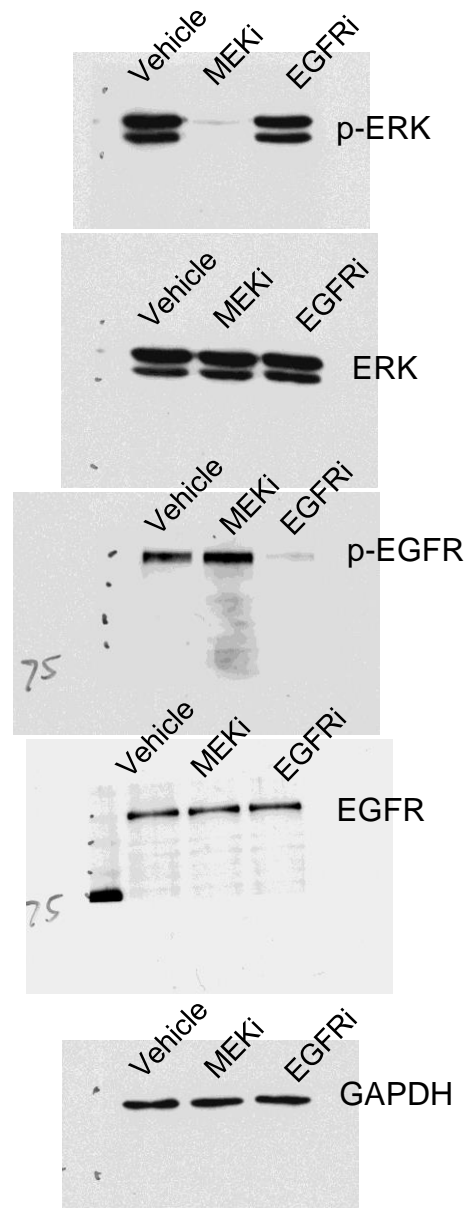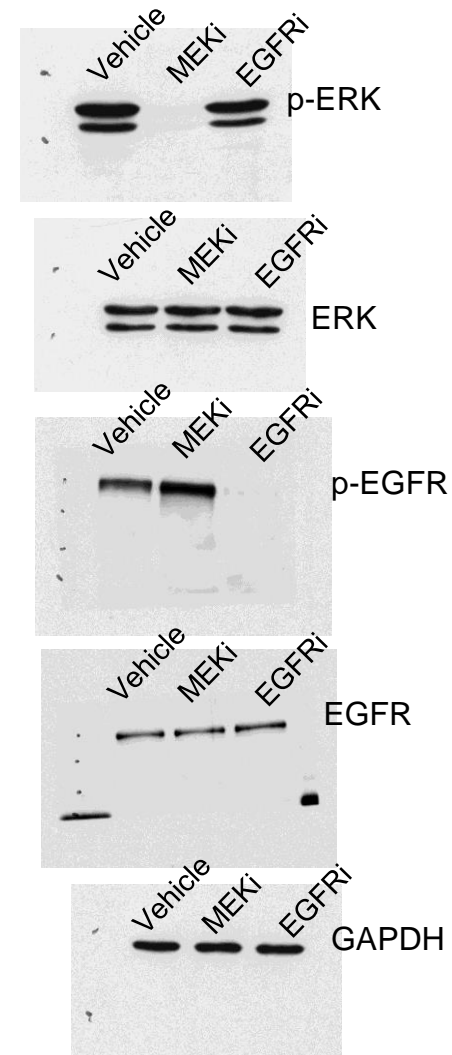

Supplement: Figure 5—source data 1. [file elife-94605-fig5-data1.zip › Figure 5-Source data 5 Uncropped and labeled gels for Figure 5.pdf]

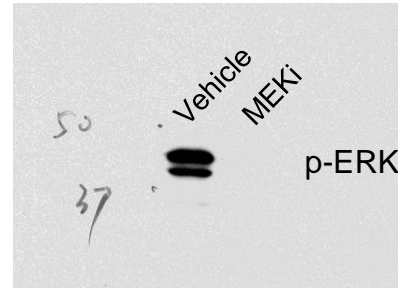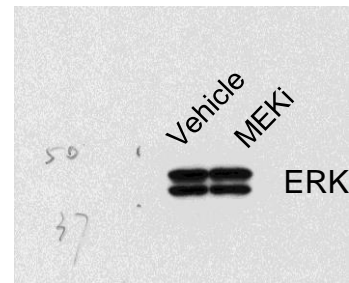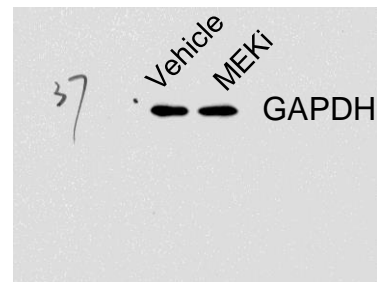

Supplement: Figure 5—source data 1. [file elife-94605-fig5-data1.zip › Figure 5-Source data 6 Uncropped and labeled gels for Figure 5.pdf]

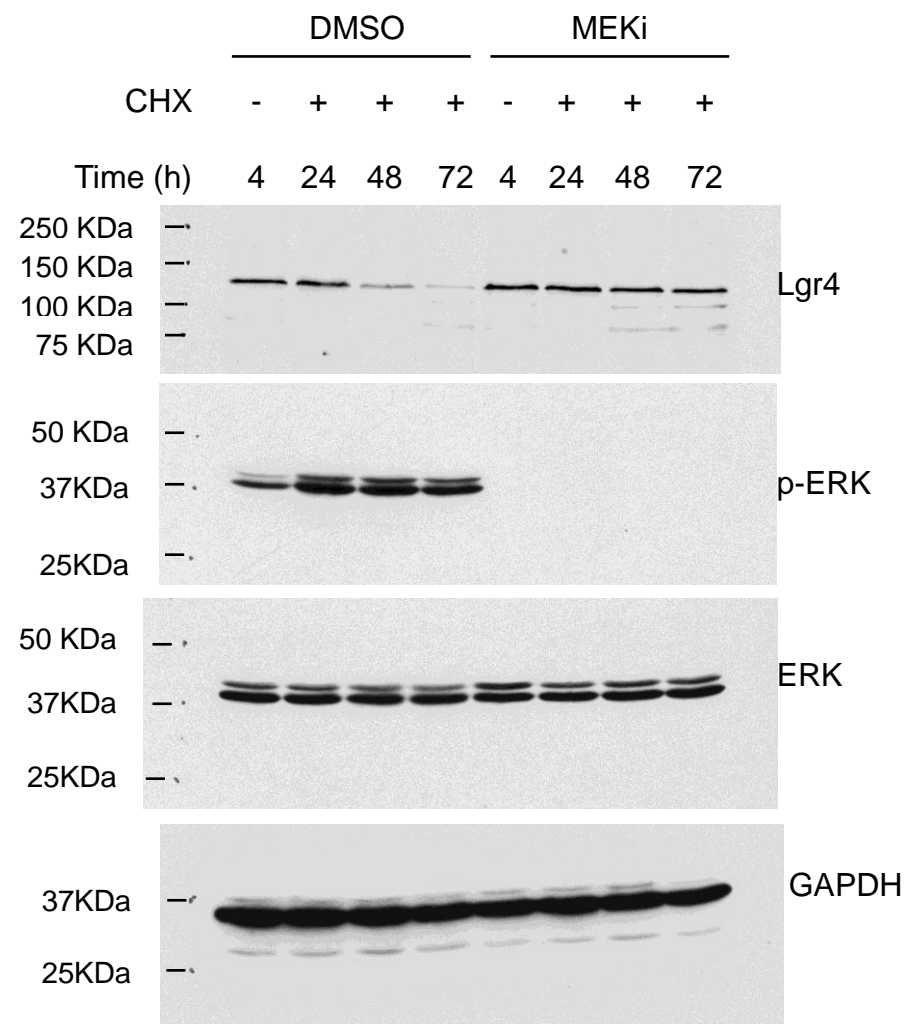

Supplement: Figure 5—source data 1. [file elife-94605-fig5-data1.zip › Figure 5-Source data 7 Uncropped and labeled gels for Figure 5.pdf]

erk

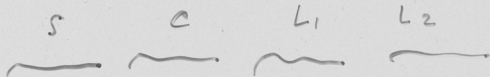

50

37

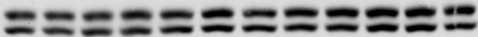

Supplement: Figure 5—source data 2. [file elife-94605-fig5-data2.zip › Figure 5-source data 1 Raw unedited gels for Figure 5.pdf]

5280  
6225

PAK

S

C

L<sub>1</sub>

L<sub>2</sub>

100

75

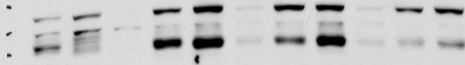

Supplement: Figure 5—source data 2. [file elife-94605-fig5-data2.zip › Figure 5-source data 2 Raw unedited gels for Figure 5.pdf]

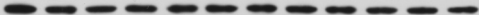

Supplement: Figure 5—source data 2. [file elife-94605-fig5-data2.zip › Figure 5-source data 3 Raw unedited gels for Figure 5.pdf]

S C L<sub>1</sub> L<sub>2</sub>

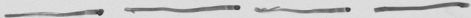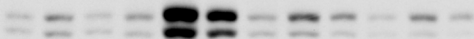

Supplement: Figure 5—source data 2. [file elife-94605-fig5-data2.zip › Figure 5-source data 4 Raw unedited gels for Figure 5.pdf]

p-estr.  
1568

12 lanes

75

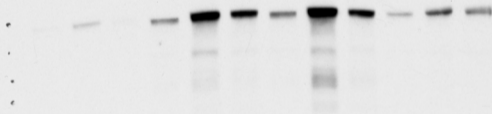

Supplement: Figure 5—source data 2. [file elife-94605-fig5-data2.zip › Figure 5-source data 5 Raw unedited gels for Figure 5.pdf]

S C L<sub>1</sub> L<sub>2</sub>

\_\_\_\_\_

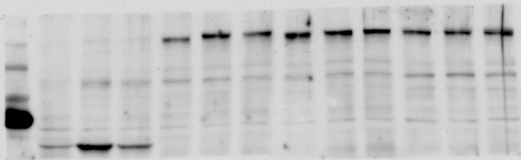

Supplement: Figure 5—source data 2. [file elife-94605-fig5-data2.zip › Figure 5-source data 7 Raw unedited gels for Figure 5.pdf]

extr

75

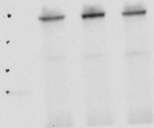

Supplement: Figure 5—source data 2. [file elife-94605-fig5-data2.zip › Figure 5-source data 9 Raw unedited gels for Figure 5.pdf]

50

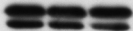

Supplement: Figure 5—source data 2. [file elife-94605-fig5-data2.zip › Figure 5-source data 10 Raw unedited gels for Figure 5.pdf]

37

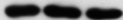

25

Supplement: Figure 5—source data 2. [file elife-94605-fig5-data2.zip › Figure 5-source data 11 Raw unedited gels for Figure 5.pdf]

50

37

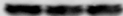

Supplement: Figure 5—source data 2. [file elife-94605-fig5-data2.zip › Figure 5-source data 12 Raw unedited gels for Figure 5.pdf]

250

.

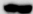

150

.

100

.

Supplement: Figure 5—source data 2. [file elife-94605-fig5-data2.zip › Figure 5-source data 14 Raw unedited gels for Figure 5.pdf]

50

57

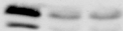

Supplement: Figure 5—source data 2. [file elife-94605-fig5-data2.zip › Figure 5-source data 15 Raw unedited gels for Figure 5.pdf]

37

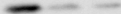

Supplement: Figure 5—source data 2. [file elife-94605-fig5-data2.zip › Figure 5-source data 16 Raw unedited gels for Figure 5.pdf]

BC + FALC 1

BC —

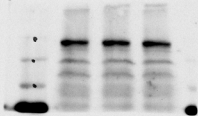

75

Supplement: Figure 5—source data 2. [file elife-94605-fig5-data2.zip › Figure 5-source data 17 Raw unedited gels for Figure 5.pdf]

erk

Bc ~~Bc~~ + FAK?

1 2

50

37

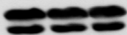

Supplement: Figure 5—source data 2. [file elife-94605-fig5-data2.zip › Figure 5-source data 18 Raw unedited gels for Figure 5.pdf]

BC BC + FAKi 4h  
1 2  

---

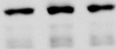

Supplement: Figure 5—source data 2. [file elife-94605-fig5-data2.zip › Figure 5-source data 19 Raw unedited gels for Figure 5.pdf]

GAPDH

BC

BC + FAKi

1 2

37

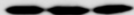

Supplement: Figure 5—source data 2. [file elife-94605-fig5-data2.zip › Figure 5-source data 20 Raw unedited gels for Figure 5.pdf]

6.14.202

(identical to  
6.13.21 1068)

p-estr 1068

BC

BC + FALC

1 2

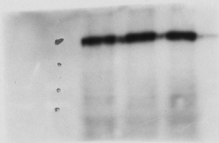

50

Supplement: Figure 5—source data 2. [file elife-94605-fig5-data2.zip › Figure 5-source data 21 Raw unedited gels for Figure 5.pdf]

p-erk

BC + F/H/C 4h  
BC 1 2  
          

37

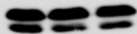

Supplement: Figure 5—source data 2. [file elife-94605-fig5-data2.zip › Figure 5-source data 22 Raw unedited gels for Figure 5.pdf]

p-FAK 397

BC

BC+FAK: 4h

1 2

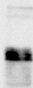

75

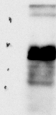

Supplement: Figure 5—source data 2. [file elife-94605-fig5-data2.zip › Figure 5-source data 23 Raw unedited gels for Figure 5.pdf]

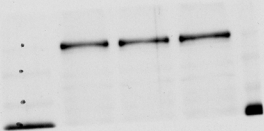

Supplement: Figure 5—source data 2. [file elife-94605-fig5-data2.zip › Figure 5-source data 24 Raw unedited gels for Figure 5.pdf]

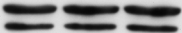

Supplement: Figure 5—source data 2. [file elife-94605-fig5-data2.zip › Figure 5-source data 25 Raw unedited gels for Figure 5.pdf]

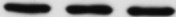

Supplement: Figure 5—source data 2. [file elife-94605-fig5-data2.zip › Figure 5-source data 26 Raw unedited gels for Figure 5.pdf]

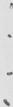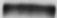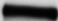

Supplement: Figure 5—source data 2. [file elife-94605-fig5-data2.zip › Figure 5-source data 27 Raw unedited gels for Figure 5.pdf]

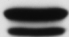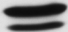

Supplement: Figure 5—source data 2. [file elife-94605-fig5-data2.zip › Figure 5-source data 28 Raw unedited gels for Figure 5.pdf]

52

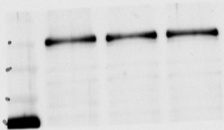

Supplement: Figure 5—source data 2. [file elife-94605-fig5-data2.zip › Figure 5-source data 29 Raw unedited gels for Figure 5.pdf]

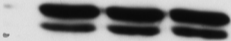

Supplement: Figure 5—source data 2. [file elife-94605-fig5-data2.zip › Figure 5-source data 30 Raw unedited gels for Figure 5.pdf]

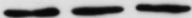

Supplement: Figure 5—source data 2. [file elife-94605-fig5-data2.zip › Figure 5-source data 31 Raw unedited gels for Figure 5.pdf]

75

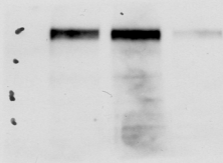

Supplement: Figure 5—source data 2. [file elife-94605-fig5-data2.zip › Figure 5-source data 32 Raw unedited gels for Figure 5.pdf]

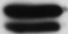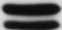

Supplement: Figure 5—source data 2. [file elife-94605-fig5-data2.zip › Figure 5-source data 33 Raw unedited gels for Figure 5.pdf]

BC BC+MEK1

ERK

50

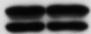

37

Supplement: Figure 5—source data 2. [file elife-94605-fig5-data2.zip › Figure 5-source data 34 Raw unedited gels for Figure 5.pdf]

BC BC + mZki

37

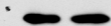

GAPDH

Supplement: Figure 5—source data 2. [file elife-94605-fig5-data2.zip › Figure 5-source data 35 Raw unedited gels for Figure 5.pdf]

p-er/c

BC BC + mZ/ci

50

37

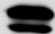

Supplement: Figure 5—source data 2. [file elife-94605-fig5-data2.zip › Figure 5-source data 36 Raw unedited gels for Figure 5.pdf]

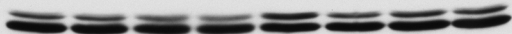

Supplement: Figure 5—source data 2. [file elife-94605-fig5-data2.zip › Figure 5-source data 37 Raw unedited gels for Figure 5.pdf]

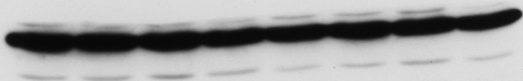

Supplement: Figure 5—source data 2. [file elife-94605-fig5-data2.zip › Figure 5-source data 38 Raw unedited gels for Figure 5.pdf]

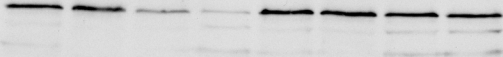

Supplement: Figure 5—source data 2. [file elife-94605-fig5-data2.zip › Figure 5-source data 39 Raw unedited gels for Figure 5.pdf]

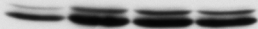

Supplement: Figure 5—source data 2. [file elife-94605-fig5-data2.zip › Figure 5-source data 40 Raw unedited gels for Figure 5.pdf]

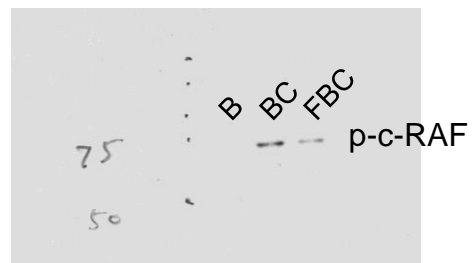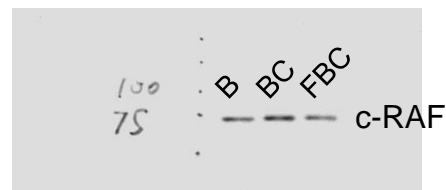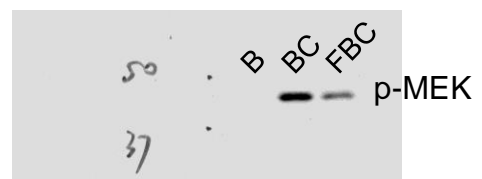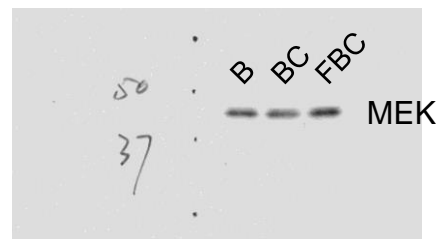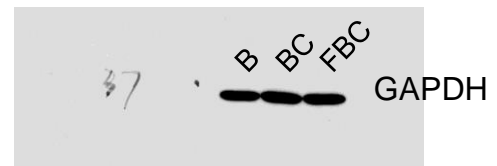

Supplement: Figure 5—figure supplement 1—source data 1. [file elife-94605-fig5-figsupp1-data1.zip › Figure 5-Figure supplement-source data 1 Uncropped and labeled gels for Figure 5-Figure supplement.pdf]

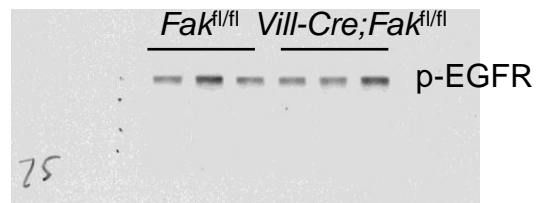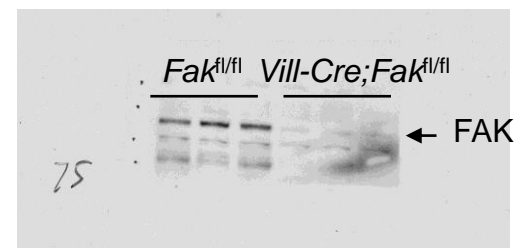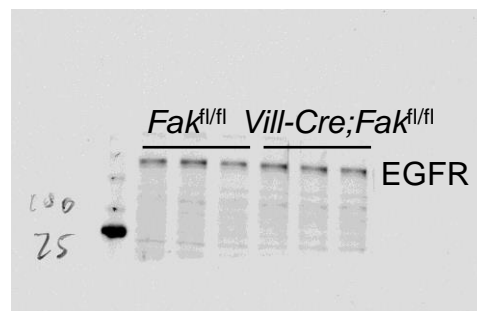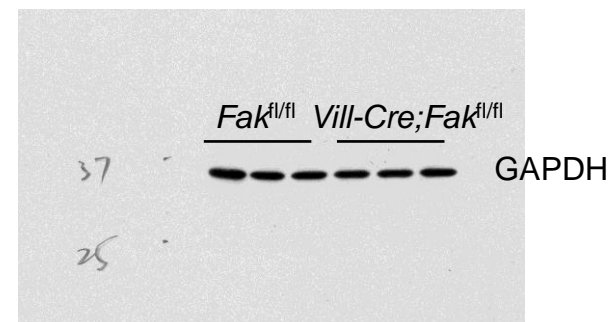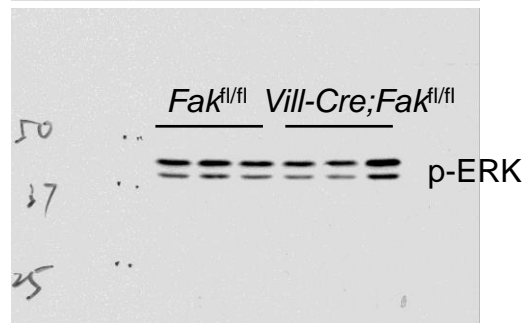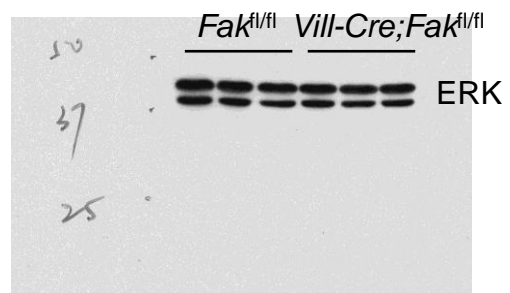

Supplement: Figure 5—figure supplement 1—source data 1. [file elife-94605-fig5-figsupp1-data1.zip › Figure 5-Figure supplement-source data 2 Uncropped and labeled gels for Figure 5-Figure supplement.pdf]

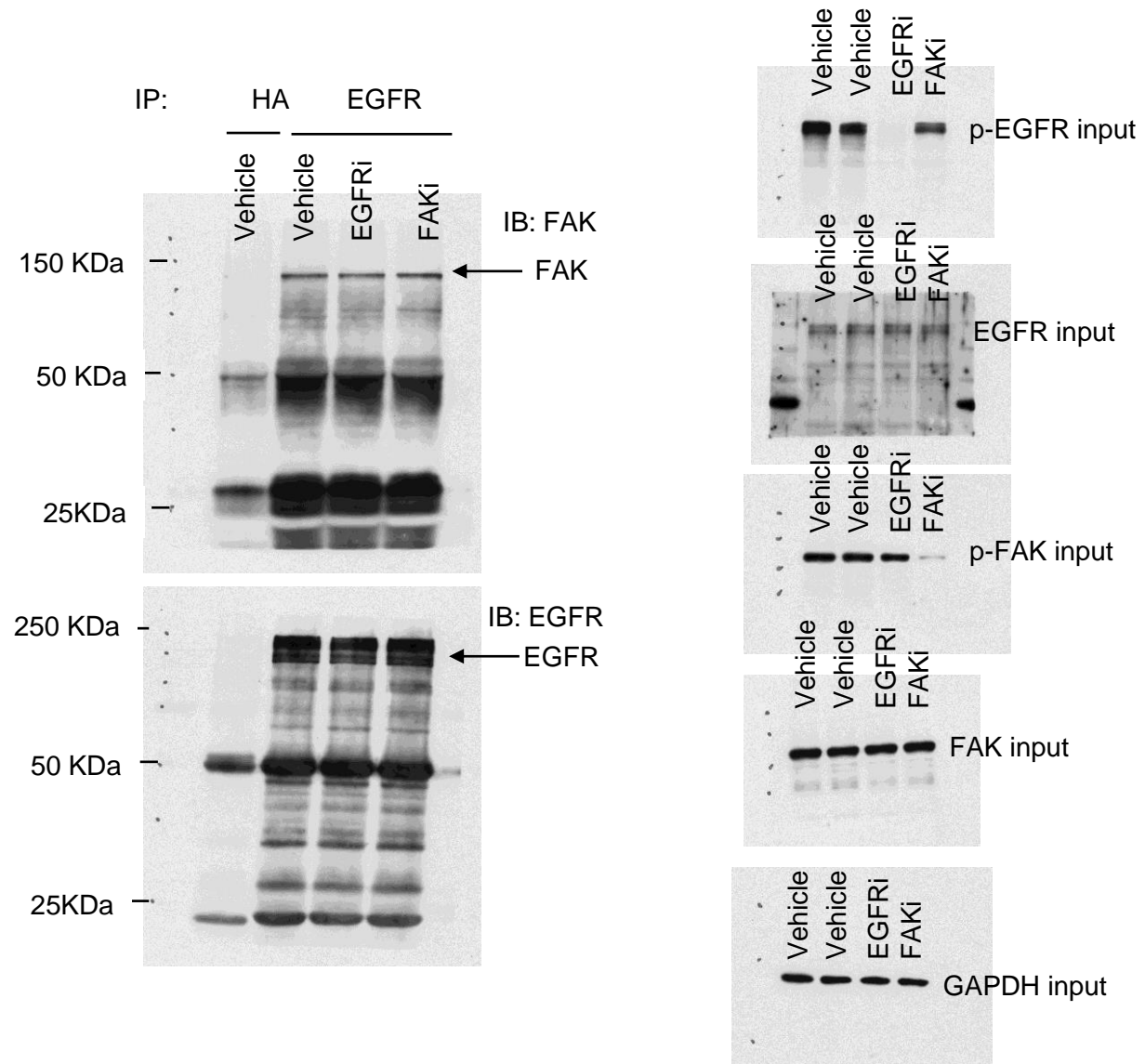

Supplement: Figure 5—figure supplement 1—source data 1. [file elife-94605-fig5-figsupp1-data1.zip › Figure 5-Figure supplement-source data 3 Uncropped and labeled gels for Figure 5-Figure supplement.pdf]

6225

GAPDH

Reblot 4-65-21

p-mgk(A)  
film

37

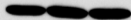

Supplement: Figure 5—figure supplement 1—source data 2. [file elife-94605-fig5-figsupp1-data2.zip › Figure 5-Figure supplement-source data 2 Raw unedited gels for Figure 5-Figure supplement.pdf]

m E/C

50

37

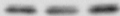

Supplement: Figure 5—figure supplement 1—source data 2. [file elife-94605-fig5-figsupp1-data2.zip › Figure 5-Figure supplement-source data 3 Raw unedited gels for Figure 5-Figure supplement.pdf]

13 BC F13C

75

50

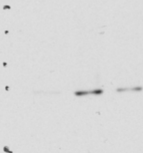

Supplement: Figure 5—figure supplement 1—source data 2. [file elife-94605-fig5-figsupp1-data2.zip › Figure 5-Figure supplement-source data 4 Raw unedited gels for Figure 5-Figure supplement.pdf]

6225

p-mE(KLA)

50

37

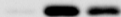

Supplement: Figure 5—figure supplement 1—source data 2. [file elife-94605-fig5-figsupp1-data2.zip › Figure 5-Figure supplement-source data 5 Raw unedited gels for Figure 5-Figure supplement.pdf]

p-mek (17)

50

37

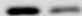

Supplement: Figure 5—figure supplement 1—source data 2. [file elife-94605-fig5-figsupp1-data2.zip › Figure 5-Figure supplement-source data 6 Raw unedited gels for Figure 5-Figure supplement.pdf]

p-est  
1068

control  
-C

FC-C

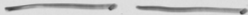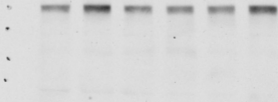

52

Supplement: Figure 5—figure supplement 1—source data 2. [file elife-94605-fig5-figsupp1-data2.zip › Figure 5-Figure supplement-source data 7 Raw unedited gels for Figure 5-Figure supplement.pdf]

100

52

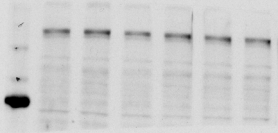

Supplement: Figure 5—figure supplement 1—source data 2. [file elife-94605-fig5-figsupp1-data2.zip › Figure 5-Figure supplement-source data 8 Raw unedited gels for Figure 5-Figure supplement.pdf]

10

37

25

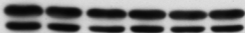

Supplement: Figure 5—figure supplement 1—source data 2. [file elife-94605-fig5-figsupp1-data2.zip › Figure 5-Figure supplement-source data 9 Raw unedited gels for Figure 5-Figure supplement.pdf]

Control

FC-C

- C

—————

—————

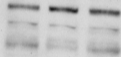

52

Supplement: Figure 5—figure supplement 1—source data 2. [file elife-94605-fig5-figsupp1-data2.zip › Figure 5-Figure supplement-source data 10 Raw unedited gels for Figure 5-Figure supplement.pdf]

37

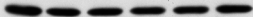

25

Supplement: Figure 5—figure supplement 1—source data 2. [file elife-94605-fig5-figsupp1-data2.zip › Figure 5-Figure supplement-source data 11 Raw unedited gels for Figure 5-Figure supplement.pdf]

50

..

37

..

25

..

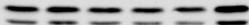

Supplement: Figure 5—figure supplement 1—source data 2. [file elife-94605-fig5-figsupp1-data2.zip › Figure 5-Figure supplement-source data 12 Raw unedited gels for Figure 5-Figure supplement.pdf]

B Bc Bc + estri 4h

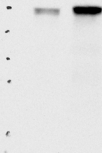

50

Supplement: Figure 5—figure supplement 1—source data 2. [file elife-94605-fig5-figsupp1-data2.zip › Figure 5-Figure supplement-source data 13 Raw unedited gels for Figure 5-Figure supplement.pdf]

total estr

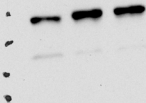

Supplement: Figure 5—figure supplement 1—source data 2. [file elife-94605-fig5-figsupp1-data2.zip › Figure 5-Figure supplement-source data 14 Raw unedited gels for Figure 5-Figure supplement.pdf]

6032

total erk (p-erk stripped  
+ reblot)

3 13c 13c+estri 4h

50

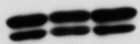

37

Supplement: Figure 5—figure supplement 1—source data 2. [file elife-94605-fig5-figsupp1-data2.zip › Figure 5-Figure supplement-source data 15 Raw unedited gels for Figure 5-Figure supplement.pdf]

PAK

B BC BC+estr

75

50

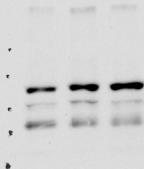

Supplement: Figure 5—figure supplement 1—source data 2. [file elife-94605-fig5-figsupp1-data2.zip › Figure 5-Figure supplement-source data 16 Raw unedited gels for Figure 5-Figure supplement.pdf]

GAPDH

13 13C 13C + ctrl

(p-mk  
stripped  
reblot)

37

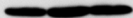

25

Supplement: Figure 5—figure supplement 1—source data 2. [file elife-94605-fig5-figsupp1-data2.zip › Figure 5-Figure supplement-source data 17 Raw unedited gels for Figure 5-Figure supplement.pdf]

21: estr cm

25: p-estr 1068  
(K)

( estr/FAHC stripped  
and  
Reblot

HA

B

BC

BC+estri

Reblot

75

25

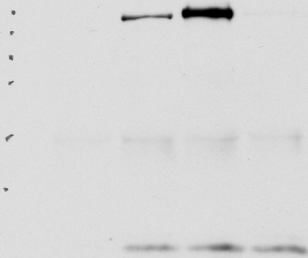

Supplement: Figure 5—figure supplement 1—source data 2. [file elife-94605-fig5-figsupp1-data2.zip › Figure 5-Figure supplement-source data 18 Raw unedited gels for Figure 5-Figure supplement.pdf]

Ip: estr

211: estr

HA<sub>13C</sub>

B 13C 13C + estr

75

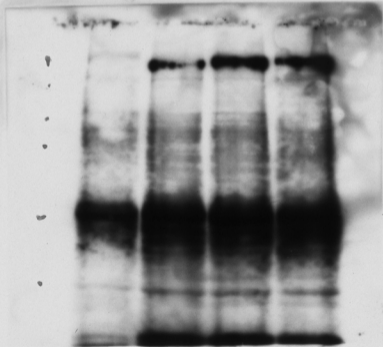

Supplement: Figure 5—figure supplement 1—source data 2. [file elife-94605-fig5-figsupp1-data2.zip › Figure 5-Figure supplement-source data 19 Raw unedited gels for Figure 5-Figure supplement.pdf]

p-erk

B BC BC+estri  
4h

5

37

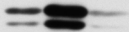

Supplement: Figure 5—figure supplement 1—source data 2. [file elife-94605-fig5-figsupp1-data2.zip › Figure 5-Figure supplement-source data 20 Raw unedited gels for Figure 5-Figure supplement.pdf]

M 1789:45  
 R 1789:45  
 1789

MH B BC BC + Estr.

25 75

Q

25

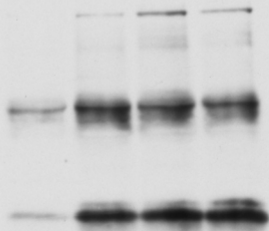

Supplement: Figure 5—figure supplement 1—source data 2. [file elife-94605-fig5-figsupp1-data2.zip › Figure 5-Figure supplement-source data 21 Raw unedited gels for Figure 5-Figure supplement.pdf]

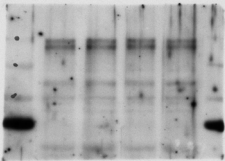

Supplement: Figure 5—figure supplement 1—source data 2. [file elife-94605-fig5-figsupp1-data2.zip › Figure 5-Figure supplement-source data 22 Raw unedited gels for Figure 5-Figure supplement.pdf]

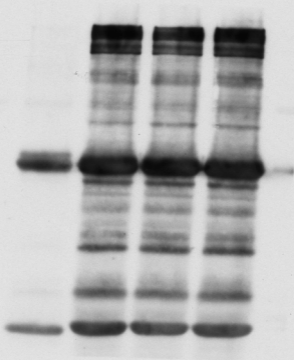

Supplement: Figure 5—figure supplement 1—source data 2. [file elife-94605-fig5-figsupp1-data2.zip › Figure 5-Figure supplement-source data 23 Raw unedited gels for Figure 5-Figure supplement.pdf]

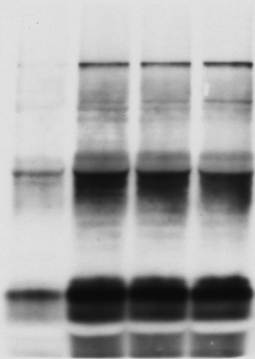

Supplement: Figure 5—figure supplement 1—source data 2. [file elife-94605-fig5-figsupp1-data2.zip › Figure 5-Figure supplement-source data 24 Raw unedited gels for Figure 5-Figure supplement.pdf]

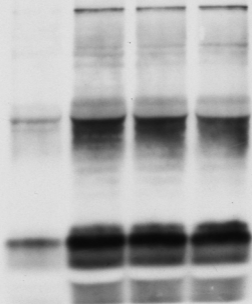

Supplement: Figure 5—figure supplement 1—source data 2. [file elife-94605-fig5-figsupp1-data2.zip › Figure 5-Figure supplement-source data 25 Raw unedited gels for Figure 5-Figure supplement.pdf]

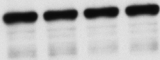

Supplement: Figure 5—figure supplement 1—source data 2. [file elife-94605-fig5-figsupp1-data2.zip › Figure 5-Figure supplement-source data 26 Raw unedited gels for Figure 5-Figure supplement.pdf]

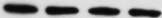

Supplement: Figure 5—figure supplement 1—source data 2. [file elife-94605-fig5-figsupp1-data2.zip › Figure 5-Figure supplement-source data 27 Raw unedited gels for Figure 5-Figure supplement.pdf]

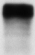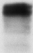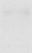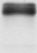

Supplement: Figure 5—figure supplement 1—source data 2. [file elife-94605-fig5-figsupp1-data2.zip › Figure 5-Figure supplement-source data 28 Raw unedited gels for Figure 5-Figure supplement.pdf]

| IP    | Flag | LGR4 |
|-------|------|------|
| HA-Ub | +    | +    |
| MEKi  | -    | -    |
| MG132 | +    | +    |

250 KDa  
150 KDa  
100 KDa  
75 KDa  
50 KDa  
37 KDa  
25KDa

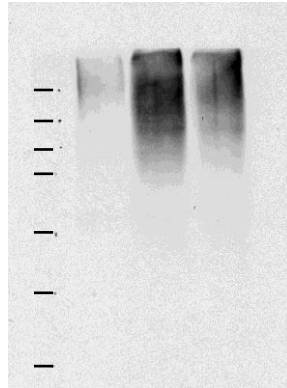

IB: Ub

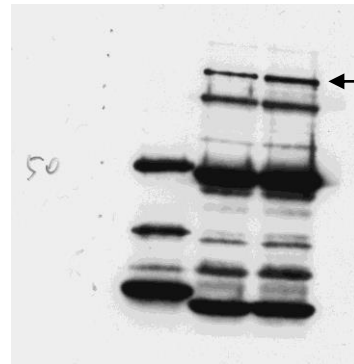

IB: LGR4

|       |   |   |   |
|-------|---|---|---|
| HA-Ub | + | + | + |
| MEKi  | - | - | + |
| MG132 | + | + | + |

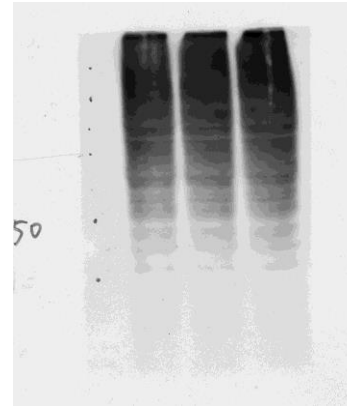

Ub input

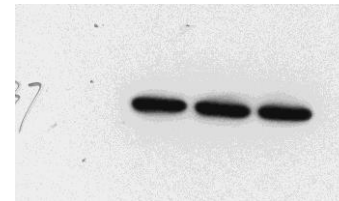

GAPDH input

Supplement: Figure 6—source data 1. [file elife-94605-fig6-data1.zip › Figure 6-Source data 4 Uncropped and labeled gels for Figure 6.pdf]

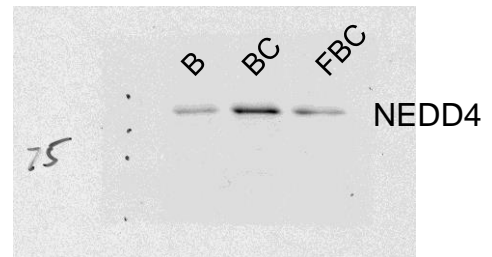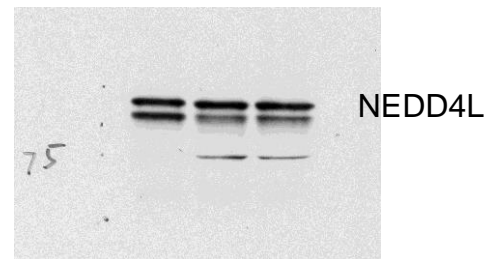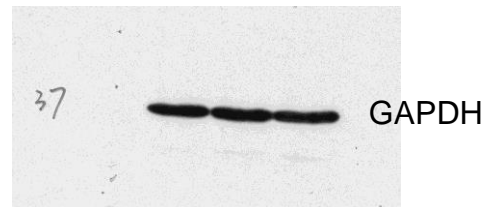

Supplement: Figure 6—source data 1. [file elife-94605-fig6-data1.zip › Figure 6-Source data 1 Uncropped and labeled gels for Figure 6.pdf]

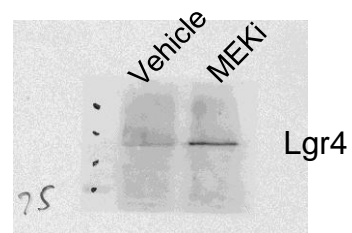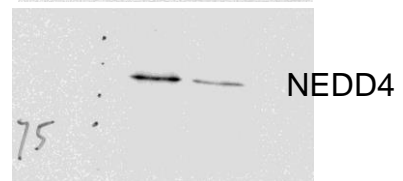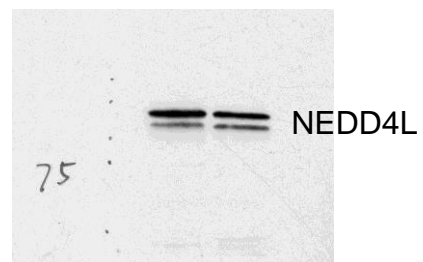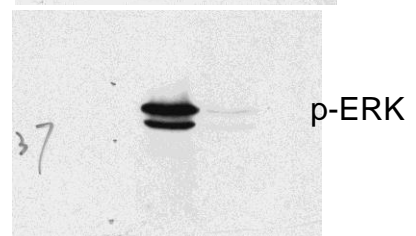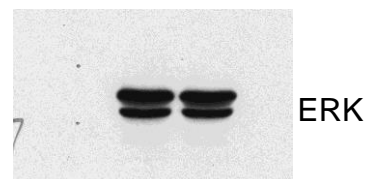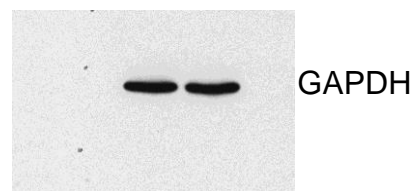

Supplement: Figure 6—source data 1. [file elife-94605-fig6-data1.zip › Figure 6-Source data 2 Uncropped and labeled gels for Figure 6.pdf]

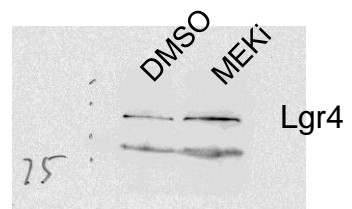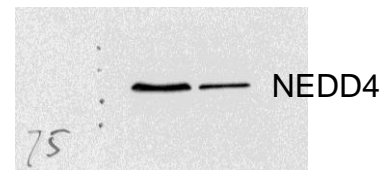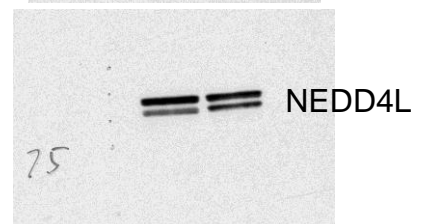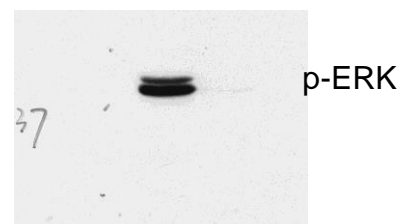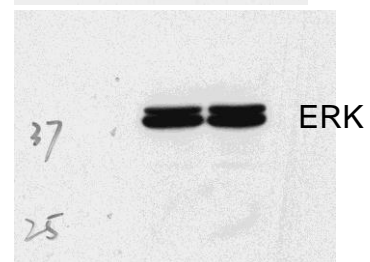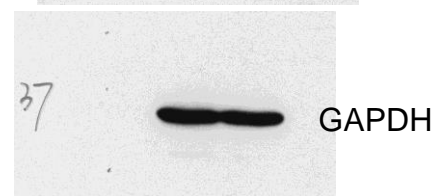

Supplement: Figure 6—source data 1. [file elife-94605-fig6-data1.zip › Figure 6-Source data 3 Uncropped and labeled gels for Figure 6.pdf]

37

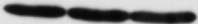

Supplement: Figure 6—source data 2. [file elife-94605-fig6-data2.zip › Figure 6-source data 1 Raw unedited gels for Figure 6.pdf]

75

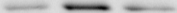

Supplement: Figure 6—source data 2. [file elife-94605-fig6-data2.zip › Figure 6-source data 2 Raw unedited gels for Figure 6.pdf]

75

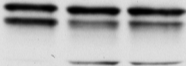

Supplement: Figure 6—source data 2. [file elife-94605-fig6-data2.zip › Figure 6-source data 3 Raw unedited gels for Figure 6.pdf]

7

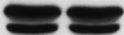

Supplement: Figure 6—source data 2. [file elife-94605-fig6-data2.zip › Figure 6-source data 4 Raw unedited gels for Figure 6.pdf]

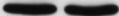

Supplement: Figure 6—source data 2. [file elife-94605-fig6-data2.zip › Figure 6-source data 5 Raw unedited gels for Figure 6.pdf]

75

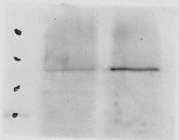

Supplement: Figure 6—source data 2. [file elife-94605-fig6-data2.zip › Figure 6-source data 6 Raw unedited gels for Figure 6.pdf]

75

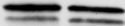

Supplement: Figure 6—source data 2. [file elife-94605-fig6-data2.zip › Figure 6-source data 7 Raw unedited gels for Figure 6.pdf]

37

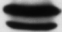

Supplement: Figure 6—source data 2. [file elife-94605-fig6-data2.zip › Figure 6-source data 9 Raw unedited gels for Figure 6.pdf]

37

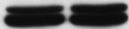

25

Supplement: Figure 6—source data 2. [file elife-94605-fig6-data2.zip › Figure 6-source data 10 Raw unedited gels for Figure 6.pdf]

37

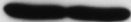

Supplement: Figure 6—source data 2. [file elife-94605-fig6-data2.zip › Figure 6-source data 11 Raw unedited gels for Figure 6.pdf]

75

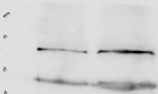

Supplement: Figure 6—source data 2. [file elife-94605-fig6-data2.zip › Figure 6-source data 12 Raw unedited gels for Figure 6.pdf]

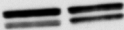

75

Supplement: Figure 6—source data 2. [file elife-94605-fig6-data2.zip › Figure 6-source data 14 Raw unedited gels for Figure 6.pdf]

37

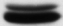

Supplement: Figure 6—source data 2. [file elife-94605-fig6-data2.zip › Figure 6-source data 15 Raw unedited gels for Figure 6.pdf]

75

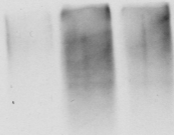

Supplement: Figure 6—source data 2. [file elife-94605-fig6-data2.zip › Figure 6-source data 16 Raw unedited gels for Figure 6.pdf]

50

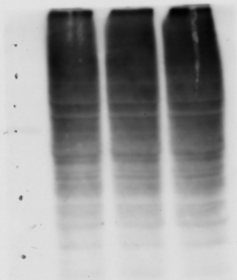

Supplement: Figure 6—source data 2. [file elife-94605-fig6-data2.zip › Figure 6-source data 17 Raw unedited gels for Figure 6.pdf]

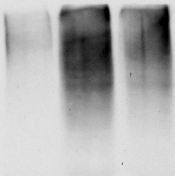

Supplement: Figure 6—source data 2. [file elife-94605-fig6-data2.zip › Figure 6-source data 18 Raw unedited gels for Figure 6.pdf]

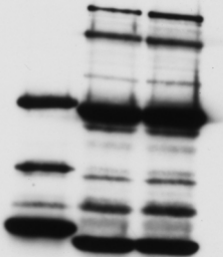

Supplement: Figure 6—source data 2. [file elife-94605-fig6-data2.zip › Figure 6-source data 19 Raw unedited gels for Figure 6.pdf]

37.

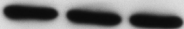

Supplement: Figure 6—source data 2. [file elife-94605-fig6-data2.zip › Figure 6-source data 20 Raw unedited gels for Figure 6.pdf]

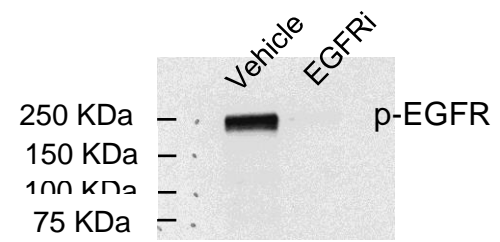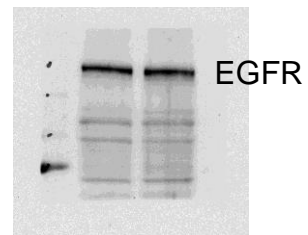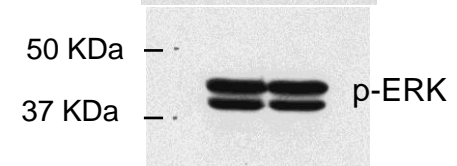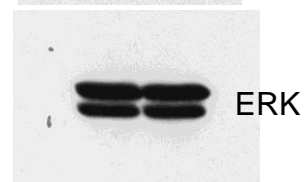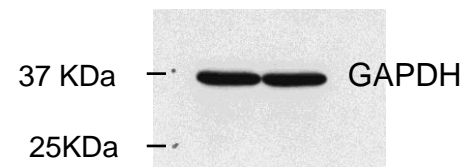

Supplement: Figure 7—source data 1. [file elife-94605-fig7-data1.zip › Figure 7-Source data 3 Uncropped and labeled gels for Figure 7.pdf]
