# Supplementary material for: FAK loss reduces BRAFV600E-induced ERK phosphorylation to promote intestinal stemness and cecal tumor formation
Source: eLife. 2024 Jun 26;13:RP94605. doi: 10.7554/eLife.94605 (PMC11208045; doi:10.7554/eLife.94605)
Supplement: Figure 5—source data 2. [file elife-94605-fig5-data2.zip › Figure 5-source data 13 Raw unedited gels for Figure 5.pdf]

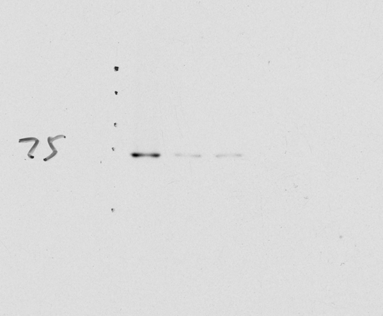
75
