# Supplementary figures and images for: FAK loss reduces BRAFV600E-induced ERK phosphorylation to promote intestinal stemness and cecal tumor formation (part 2 of 2)
Source: eLife. 2024 Jun 26;13:RP94605. doi: 10.7554/eLife.94605 (PMC11208045; doi:10.7554/eLife.94605)

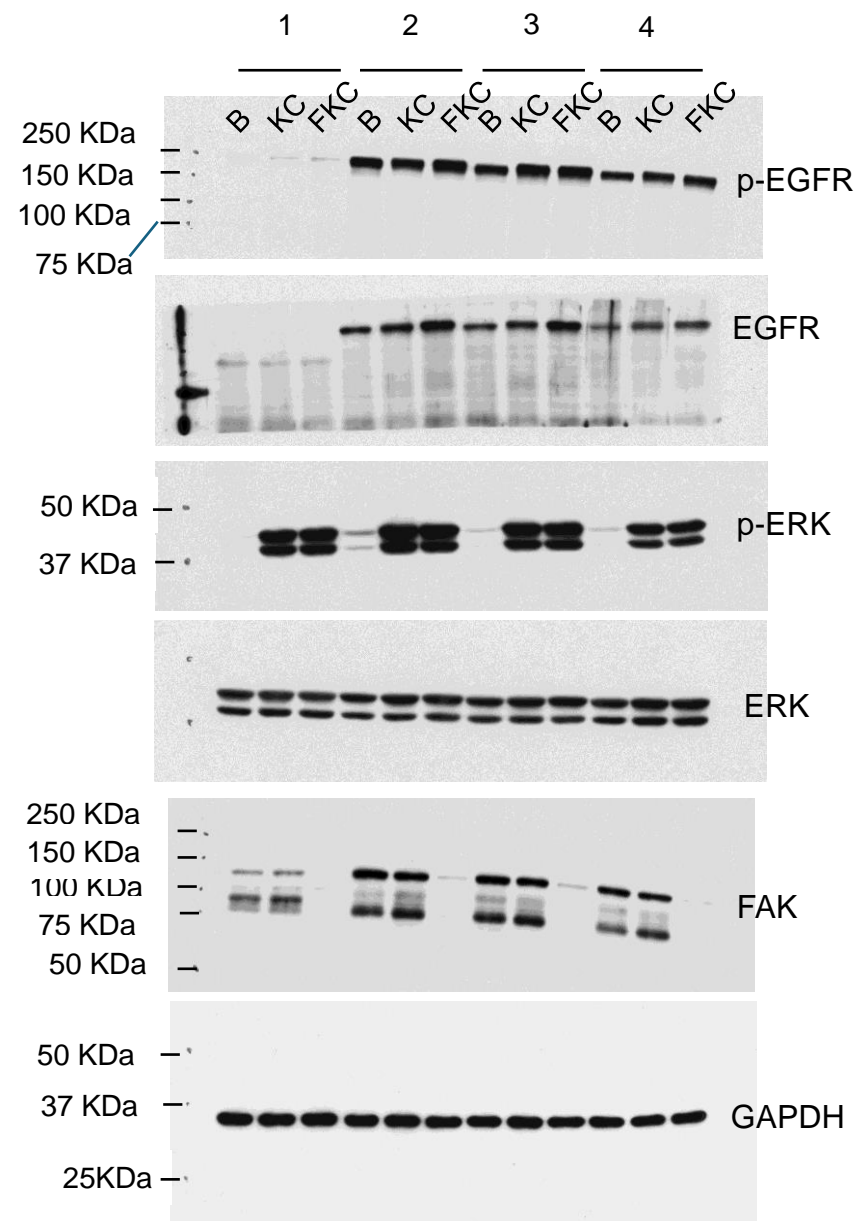

Supplement: Figure 7—source data 1. [file elife-94605-fig7-data1.zip › Figure 7-Source data 1 Uncropped and labeled gels for Figure 7.pdf]

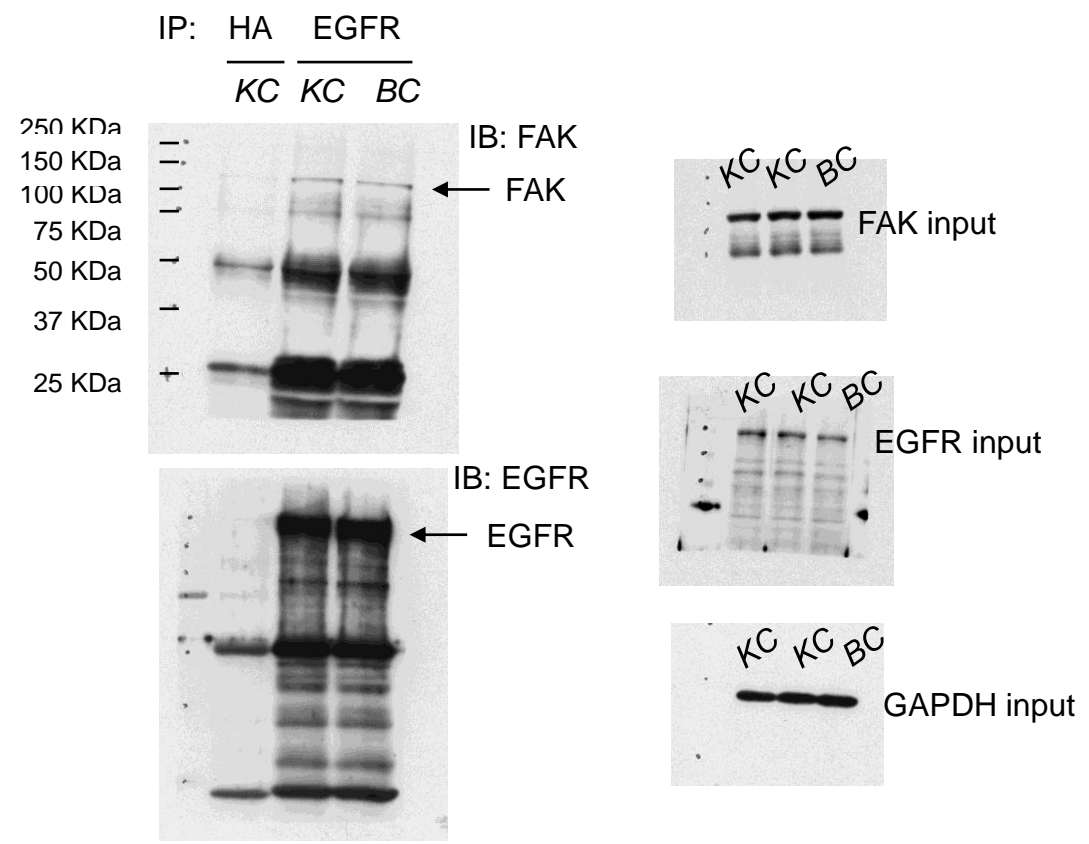

Supplement: Figure 7—source data 1. [file elife-94605-fig7-data1.zip › Figure 7-Source data 2 Uncropped and labeled gels for Figure 7.pdf]

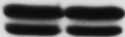

Supplement: Figure 7—source data 2. [file elife-94605-fig7-data2.zip › Figure 7-source data 16 Raw unedited gels for Figure 7.pdf]

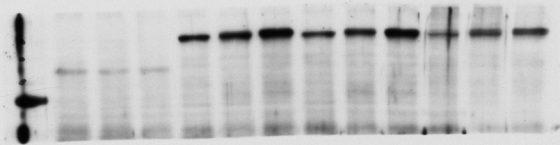

Supplement: Figure 7—source data 2. [file elife-94605-fig7-data2.zip › Figure 7-source data 1 Raw unedited gels for Figure 7.pdf]

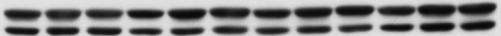

Supplement: Figure 7—source data 2. [file elife-94605-fig7-data2.zip › Figure 7-source data 2 Raw unedited gels for Figure 7.pdf]

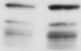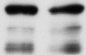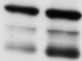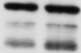

Supplement: Figure 7—source data 2. [file elife-94605-fig7-data2.zip › Figure 7-source data 3 Raw unedited gels for Figure 7.pdf]

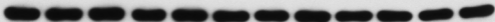

Supplement: Figure 7—source data 2. [file elife-94605-fig7-data2.zip › Figure 7-source data 4 Raw unedited gels for Figure 7.pdf]

1  
2  
3  
4

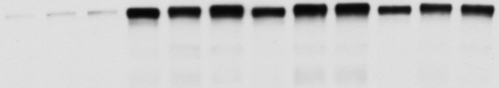

Supplement: Figure 7—source data 2. [file elife-94605-fig7-data2.zip › Figure 7-source data 5 Raw unedited gels for Figure 7.pdf]

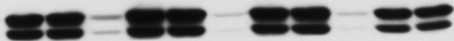

Supplement: Figure 7—source data 2. [file elife-94605-fig7-data2.zip › Figure 7-source data 6 Raw unedited gels for Figure 7.pdf]

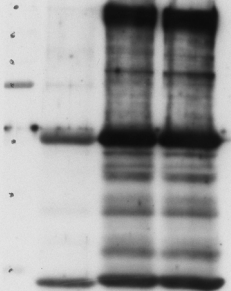

Supplement: Figure 7—source data 2. [file elife-94605-fig7-data2.zip › Figure 7-source data 7 Raw unedited gels for Figure 7.pdf]

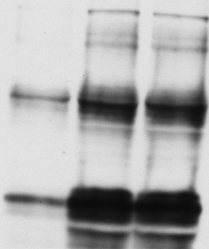

Supplement: Figure 7—source data 2. [file elife-94605-fig7-data2.zip › Figure 7-source data 8 Raw unedited gels for Figure 7.pdf]

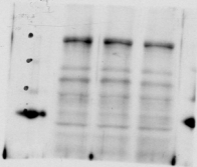

Supplement: Figure 7—source data 2. [file elife-94605-fig7-data2.zip › Figure 7-source data 9 Raw unedited gels for Figure 7.pdf]

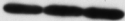

Supplement: Figure 7—source data 2. [file elife-94605-fig7-data2.zip › Figure 7-source data 10 Raw unedited gels for Figure 7.pdf]

•

•

•

•

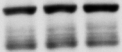

Supplement: Figure 7—source data 2. [file elife-94605-fig7-data2.zip › Figure 7-source data 11 Raw unedited gels for Figure 7.pdf]

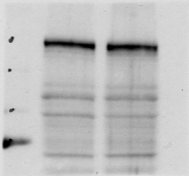

Supplement: Figure 7—source data 2. [file elife-94605-fig7-data2.zip › Figure 7-source data 12 Raw unedited gels for Figure 7.pdf]

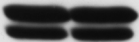

Supplement: Figure 7—source data 2. [file elife-94605-fig7-data2.zip › Figure 7-source data 13 Raw unedited gels for Figure 7.pdf]

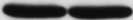

Supplement: Figure 7—source data 2. [file elife-94605-fig7-data2.zip › Figure 7-source data 14 Raw unedited gels for Figure 7.pdf]

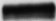

Supplement: Figure 7—source data 2. [file elife-94605-fig7-data2.zip › Figure 7-source data 15 Raw unedited gels for Figure 7.pdf]
