# Supplementary material for: FAK loss reduces BRAFV600E-induced ERK phosphorylation to promote intestinal stemness and cecal tumor formation
Source: eLife. 2024 Jun 26;13:RP94605. doi: 10.7554/eLife.94605 (PMC11208045; doi:10.7554/eLife.94605)
Supplement: Supplementary file 2. [file elife-94605-supp2.docx]

Supplementary Table 2. Antibody used in this study

| **Name** | **Company** | **Catalog nubmer** | **Usage** |
| --- | --- | --- | --- |
| Anti-FAK | Cell Signaling Technology | 3285 | IHC, IB |
| Anti-p-FAK^Y397^ | Cell Signaling Technology | 3283 | IB |
| Anti-p-ERK1/2^T202/Y204^ | Cell Signaling Technology | 4370 | IB |
| Anti-ERK1/2 | Cell Signaling Technology | 4695 | IB |
| Anti-p-EGFR^Y1068^ | Cell Signaling Technology | 2234 | IB |
| Anti-p-c-RAF^S338^ | Cell Signaling Technology | 9427 | IB |
| Anti-c-RAF | Cell Signaling Technology | 9422 | IB |
| Anti-p-MEK1/2^S217/221^ | Cell Signaling Technology | 9154 | IB |
| Anti-MEK1/2 | Cell Signaling Technology | 9122 | IB |
| Anti-GAPDH | Cell Signaling Technology | 2118 | IB |
| Anti-NEDD4 | Cell Signaling Technology | 2740 | IB |
| Anti-NEDD4L | Cell Signaling Technology | 4013 | IB |
| Anti-Ubiquitin | Cell Signaling Technology | 20326 | IB |
| Anti-EGFR | Santa Cruz Biotechnology | sc-373746 | IB |
| Anti-EGFR agarose | Santa Cruz Biotechnology | sc-373746 AC | IP |
| Anti-LGR4 | Santa Cruz Biotechnology | sc-390630 | IB |
| Anti-LGR4 agarose | Santa Cruz Biotechnology | sc-390630 AC | IP |
| Anti-BrdU | MilloporeSigma | B8434 | IHC |
| Anti-MPO | Abcam | ab208670 | IHC |
| Anti-RFP | Rockland Immunochemicals | 600-401-379 | IHC |

IHC, immunohistochemistry; IB, immunoblotting; IP, immunoprecipitation
