# Supplementary material for: FAK loss reduces BRAFV600E-induced ERK phosphorylation to promote intestinal stemness and cecal tumor formation
Source: eLife. 2024 Jun 26;13:RP94605. doi: 10.7554/eLife.94605 (PMC11208045; doi:10.7554/eLife.94605)
Supplement: Supplementary file 3. [file elife-94605-supp3.docx]

| **Gene name** | **Forward (5’-3’)** | **Reverse (5’-3’)** |
| --- | --- | --- |
| Mouse *β-actin* | CATTGCTGACAGGATGCAGAAGG | TGCTGGAAGGTGGACAGTGAGG |
| *Dusp4* | CGAGTACATCGACGCAGTAAA | ACCCGCTTCTTCATCATCAG |
| *Dusp6* | GAATGAGAACACTGGTGGAGAG | GGAACTTACTGAAGCCACCTT |
| *Etv4* | GGTGATGGAGTGATGGGTTATG | CTTCCTGCTTGATGTCTCCTTC |
| *Etv5* | CTACATGAGAGGCGGGTATTTC | GGTACCACGCAAGTATCATCAA |
| *Phlda1* | CTTCACTGTGGTGATGACGGAG | TTCTGCCTGGTAGACTTGACCG |
| *Phlda2* | TTCACCATCGTCACCAACTATTA | CGGTTCTGGAAGTCGATCAG |
| *Spry4* | CCGCTGTGACCAGGATATTAC | TGGAGCCATGTGATCTAGGA |
| *Ccnd1* | CAGAGGCGGATGAGAACAAG | GAGGGTGGGTTGGAAATGAA |
| *Egr1* | AACAACCCTATGAGCACCTG | GAGTCGTTTGGCTGGGATAA |
| *Fosl1* | ACACCCTCTCTGACTCCTTT | CACTGCTGCTGCTACTCTTT |
| *Myc* | TCGCTGCTGTCCTCCGAGTCC | GGTTTGCCTCTTCTCCACAGAC |
| *Lgr5* | GAGTCAACCCAAGCCTTAGTATCC | CATGGGACAAATGCAACTGAAG |
| *Lgr4* | GTCCTAACCCTCCAGAACAATC | CCTCCGGGACTGAGGTAATA |
| Human *β-ACTIN* | CACTCTTCCAGCCTTCCTTC | GGATGTCCACGTCACACTTC |
| Human *LGR4* | GCATCCCTGACTTTGCATTTAC | AGGTCTCCAGGTTATCTAGTCC |

Supplementary Table 3. PCR primers used in this study
